# Economics of Obesity — Learning from the Past to Contribute to a Better Future

**DOI:** 10.3390/ijerph110404007

**Published:** 2014-04-14

**Authors:** Jaithri Ananthapavan, Gary Sacks, Marj Moodie, Rob Carter

**Affiliations:** 1 Deakin Health Economics, Faculty of Health, Deakin University, 221 Burwood Highway, Burwood, Victoria 3125, Australia; E-Mails: marj.moodie@deakin.edu.au (M.M.); rob.carter@deakin.edu.au (R.C.); 2 WHO Collaborating Centre for Obesity Prevention, Faculty of Health, Deakin University, 221 Burwood Highway, Burwood, Victoria 3125, Australia; E-Mail: gary.sacks@deakin.edu.au

**Keywords:** obesity, prevention, economic evaluation, priority setting, interventions

## Abstract

The discipline of economics plays a varied role in informing the understanding of the problem of obesity and the impact of different interventions aimed at addressing it. This paper discusses the causes of the obesity epidemic from an economics perspective, and outlines various justifications for government intervention in this area. The paper then focuses on the potential contribution of health economics in supporting resource allocation decision making for obesity prevention/treatment. Although economic evaluations of single interventions provide useful information, evaluations undertaken as part of a priority setting exercise provide the greatest scope for influencing decision making. A review of several priority setting examples in obesity prevention/treatment indicates that policy (as compared with program-based) interventions, targeted at prevention (as compared with treatment) and focused “upstream” on the food environment, are likely to be the most cost-effective options for change. However, in order to further support decision makers, several methodological advances are required. These include the incorporation of intervention costs/benefits outside the health sector, the addressing of equity impacts, and the increased engagement of decision makers in the priority setting process.

## 1. Introduction

The global obesity epidemic and its impact on global morbidity and mortality have been well reported [1,2,3]. Overweight and obesity have been steadily increasing globally over the last 30 years, and in many countries, such as Australia, elevated Body Mass Index (BMI) has overtaken high blood pressure and smoking to become the leading risk factor contributing to the burden of disease (responsible for 8.3% of the total Australian disease burden in 2010) [4].

The goals of managing the obesity epidemic and potentially reversing it are hinged on interdisciplinary collaborative efforts in research, policy development and intervention implementation by professionals from diverse disciplines and service sectors. One such discipline is health economics, which plays a varied role in both understanding the problem of obesity and in evaluating efforts to treat and prevent it. The aims of this paper are to review the potential contribution of health economics in understanding the problem of obesity and possible solutions. We also aim to outline the lessons learnt from priority setting studies in this area, review the gaps in the cost-effectiveness evidence base, and suggest areas for future research.

## 2. Overview of the Economics of Obesity

Whilst obesity can be viewed as a simple imbalance between caloric intake and expenditure, several authors have drawn on theoretical and empirical economic evidence to explain both the individual choices leading to a caloric surplus and the environmental factors that encourage such choices [5,6,7,8]. Economic growth, a fundamental macro-economic objective, has been described as a systemic driver of the obesity epidemic [1,9]. The quest for sustained and higher levels of economic growth is underpinned by increased consumption of goods and services; including food, beverages and energy saving devices. Eggers and colleagues [10] argue that although economic growth is a key factor which lifts low income countries out of poverty and is positively correlated with improved population health, a country’s gross domestic product (GDP) ultimately appears to increase beyond the optimal level (“sweet spot”) and produces diminishing marginal returns on health. Economic growth beyond the presumed sweet spot is hypothesised to result in the transition from populations experiencing high mortality associated with infectious (communicable) diseases to mortality and morbidity associated with chronic (and non-communicable) disease caused by overconsumption [9,10].

Similarly, while the notion of “consumer sovereignty” and the essential role of markets pervades orthodox economic thinking (*i.e*., individuals are well placed to decide on the food they consume and their physical activity), it is now increasingly recognised that people are driven to become more overweight and obese as a result of the obesogenic environment in which they live [1,6,7]. The increased production of cheap, tasty, energy-dense food, together with improved food distribution and highly pervasive and persuasive marketing, creates a “push effect” that drives over consumption of calories [1]. In this highly complex food environment, many of the choices people make are outside individual awareness leading people to consume excess energy without even realising it (so called, passive overconsumption) [11]. For physical activity, the environment has been described as a moderator which acts to amplify or attenuate the impact of the obesity drivers [1]. Urbanisation, built environments and technological advances have changed the nature of workplaces, the transport system, and the opportunities for physical activity. In addition, the increased opportunity cost associated with undertaking physical activity during scarce leisure time has contributed to increasingly sedentary lifestyles [1,7,8]. Thus with regard to both food choices and physical activity, it is unlikely that the obesity epidemic is due to informed individual preference. It follows therefore, that unless there are fundamental changes to the obesogenic environment, the epidemic is unlikely to diminish. This takes us to economic thinking about the basis for government intervention.

There is evidence that the level of obesity varies amongst market-based capitalist countries, and that those with greater government regulation of markets, higher social welfare spending and more equitable distribution of wealth generally benefit from lower levels of obesity prevalence [10]. Whilst such “ecological” data is interesting, most economists would nonetheless want to see the economic rationale for government intervention. Economic reasoning would focus on the case for “market failure”, together with the existence of any evidence to suggest that government intervention would be effective and not result in “government failure”. Examples of market failure include imperfect information, externalities (impacts not internalised in market prices), monopoly power and irrational individual behaviour [12]. There is evidence that all of these factors exist in the case of obesity. The ubiquitous marketing environment that influences human behaviour in order to achieve higher company profits, makes it difficult for individuals to make optimal choices in terms of their health [7,12]. In Australia in 2005, the total annual healthcare cost of overweight and obesity was estimated to be approximately AUD 21 billion [13]. These significant healthcare costs are a “negative externality” as the costs are borne by everyone in society through the national universal tax funded health insurance scheme and are not limited to those who are themselves overweight or obese. Another potential justification for government intervention is to protect consumers who act irrationally by prioritising short term gratification from excess calorie consumption, over longer term impacts on health [1,5,12]. However, as described above, it is more likely that over-consumption is driven by environmental factors than irrational behaviour. The economic rationale for government intervention is stronger in relation to interventions aimed at preventing obesity in children who make consumption choices despite the high level of information asymmetry and their diminished ability to consider the future consequences of their poor food choices [1,5,12]. Hale and colleagues [14] also make an economic case for government intervention for prevention and health promotion activities based on the fact that health information is a “public good” and is therefore likely to be undersupplied by the market.

Establishing the case for market failure is a “necessary but not sufficient condition” from an economic perspective to justify government action. Economists have long been concerned that in intervening, governments may distort markets, have unintended consequences, or that their actions may simply not work. The development of the decision sciences, including economic evaluation, can be traced to this concern to assist policy formation. In the case of economic appraisal, the rationale is to establish that government intervention constitutes “value-for-money” in the use of limited government resources. The notion of “value” is the central construct here. The definition and measurement of value is both a technical matter (which form of economic appraisal to use) and an important ethical matter. Over and above matters of market failure, governments are conscious of the “rights of citizenship” and often take action to ensure access to services that economists call “merit goods”, such as education and health care. In fact, many economists would concede that governments are more likely to intervene initially on the basis of social justice concerns, and then use market failure/government failure arguments to sort out the detail. Such social justice concerns become more compelling when the protection of vulnerable members of society are involved. This is evidenced by public support for government intervention to address the growing rates of obesity amongst children [15,16,17].

## 3. The Contribution of Health Economics

If government intervention to change the obesogenic environment is justified, how can the discipline of health economics make a significant contribution? Aiming to address the question of how to allocate scarce resources to maximise society’s welfare, economists undertake studies that describe, predict, explain and evaluate the current situation and possible alternatives [18]. Cost of illness (COI) studies describe the economic costs of obesity by estimating the healthcare costs and costs of lost productivity attributable to obesity. Over the last 20 years, the volume of cost of obesity publications has grown and there are now several reviews of such studies in specific populations, such as subgroups of different ages, sex, socio-economic status (SES) and co-morbidities [19,20,21,22]. Burden of disease (BoD) and COI studies are useful in quantifying the disease and cost burden of obesity and, therefore, highlighting the need for action. However, there are several limitations to COI/BoD studies that generally preclude them from providing sufficient evidence to support resource allocation decision making, largely due to their focus on the cost or health burden of disease, rather than on comparing potential solutions [21]. Therefore, unless integrated with economic evaluations of the options for change, the role of COI/BoD studies is limited to describing the problem of obesity, and provides little guidance on solutions to the problem [19,21].

A solutions-based approach to obesity prevention and treatment (referred to as obesity management hereafter) requires high quality, full economic evaluation studies which use explicit methods to inform which interventions are both effective and offer value-for-money [18]. Economic evaluations provide answers to the policy question—what are the additional benefits of funding an intervention relative to its costs. The results are expressed as the net cost per unit of benefit. Results from full economic evaluations allow decision makers to make an informed judgement based on the incremental cost of implementing the intervention relative to the potential benefits foregone by maintaining the status quo [23,24].

The availability of economic evaluation evidence to guide obesity policy is varied. Certain clinical interventions such as pharmaceutical management of obesity and bariatric surgery have been the topic of several economic evaluations with reviews reporting over twenty-seven economic evaluations of these interventions [25,26]. However there are other categories of obesity interventions that have had little attention in terms of evaluation. A recent systematic review of obesity management interventions aimed at pre-school children found that there were no economic evaluations published covering this topic [27]. A review of economic evaluations of fiscal policies to prevent obesity found that only three full economic evaluations have been undertaken in recent years [28].

There are several reasons for the paucity of economic evaluations for some types of obesity interventions and abundance in others. Firstly, an essential component of an economic evaluation is the evidence base around an intervention’s effectiveness. Interventions aimed at treating obesity through the use of pharmaceuticals or bariatric surgery are more suitable for evaluation using gold standard methods such as randomised controlled trials (RCTs). In contrast, policy interventions such as fiscal measures are complex and trial-based studies are less feasible to conduct [6]. Treatment options are also better positioned to demonstrate efficacy results with short to medium term follow up. On the other hand, interventions aimed at obesity prevention are often more complex due to the contribution of the multiple causal factors and are likely to take much longer to show benefits. Therefore short term summative evaluations, undertaken before the intervention has “matured” will not adequately demonstrate their true effectiveness [29]; and hence are less likely to be published [30].

Another reason for the relatively large volume of literature pertaining to clinical interventions is possibly related to the commercial interest of publishing the positive results of certain treatment options such as pharmaceuticals and medical devices used in obesity surgery. Industry sponsored studies have stringent requirements for approval and therefore commercial success, hence these studies are also more likely to have higher budgets and may be of superior design quality [31].

## 4. Priority Setting Obesity Interventions—What is the Current Evidence?

Single intervention evaluations provide information to decision makers faced with one choice—to fund or modify a specific intervention or maintain the status quo. However, they provide little insight into the prioritising of a suite of potential interventions. For example, Australian decision makers tasked with allocating scarce resources with the aim of reducing obesity prevalence are unlikely to be able to make a decision of whether or not to publicly fund bariatric surgery based on the cost-effectiveness results of bariatric surgery compared to no surgery. Whilst cost-effectiveness results of a single intervention are useful, decision makers are faced with a myriad of other questions: are the results transferable from the evaluation setting to the Australian healthcare setting; will the cost-effectiveness results change when the trial based intervention is scaled up to the population level; are there other interventions, such as community prevention interventions, that are more cost-effective than bariatric surgery at the population level; and will the decision to fund bariatric surgery be politically and practically feasible to implement? Single intervention evaluations are therefore limited in their ability to inform priority setting decision making [32].

In order to make an impact on the strategic direction of policy related to population obesity management, decision makers require a systematic and explicit approach to priority setting which is fair and evidence-based [33]. Health economics can make a significant contribution to this through the conduct of studies that provide data on the cost-effectiveness of a suite of interventions which have been evaluated using consistent methods within the same decision making context. The technical results of such evaluations need to be considered within a decision-making framework which explicitly considers other factors of importance to decision makers such as “equity” and “feasibility of implementation”. There is no “gold standard” when it comes to priority setting methodology and several approaches have been trialed [32,33,34,35]. The following section reviews evidence from four priority setting exercises related to obesity management—Assessing Cost-Effectiveness (ACE) in Obesity, ACE—Prevention, a component of the Pacific Obesity Prevention in Communities (OPIC) project and a joint initiative by the Organisation for Economic Co-operation and Development (OECD) and the World Health Organisation (WHO).

The ACE methodology [32] has been used in ACE‒Obesity and ACE‒Prevention, which are examples of priority setting studies related to obesity management in the Australian context. The methods and results from these studies have been published elsewhere [36,37,38,39,40,41,42,43,44,45,46,47,48]. ACE-Obesity and ACE-Prevention were the first studies in Australia to conduct a systematic evaluation of obesity interventions within a priority setting context with the specific aim of providing the relevant information required by government decision makers [36,38,39]. The ACE methodology aims to combine the two main requirements for policy decision making and priority setting—the application of decision rules with a focus on technical rigour, together with due process, where rational decisions are made following debate and consensus building amongst key stakeholders [32,37]. A key characteristic of the ACE methodology is the use of a standardised evaluation protocol (common reference year, perspective, timeframe, target population and methods for measuring costs and benefits, *etc.*) for all interventions [32,37]. Other key aspects of the ACE methodology are the inclusion of a representative group of stakeholders who are involved in all aspects of the project, and the two-stage assessment of benefit where the technical cost-effectiveness results are considered alongside other considerations (referred to as “second-stage filters”) of importance to decision makers (such as equity, feasibility of implementation, affordability, acceptability, sustainability, strength of evidence and potential for side effects) [32,36].

The ACE-Obesity study evaluated the 13 most relevant interventions to government decision makers related to obesity management in children and adolescents [36]. The ACE-Prevention study investigated 150 interventions aimed primarily at preventing (127 interventions) but also treating (23 interventions) non-communicable diseases. Of these 150 interventions, nine interventions related to obesity management [39]. A review of the outcomes from the ACE studies can help generate knowledge of the characteristics of obesity management interventions that determine their cost-effectiveness profile. The main findings are outlined below in Table 1, Table 2 and Table 3. In accordance with the commonly used benchmark in Australia, a decision threshold of “AUD 50,000 per disability adjusted life year (DALY) averted” was used to determine the cost-effectiveness of interventions.

### 4.1. Prevention vs. Treatment

There were eleven treatment/targeted secondary prevention interventions investigated in the ACE studies, with over half of them being cost-effective. Whilst targeted treatment was shown to be relatively costly, it was effective in inducing weight loss. There were also two interventions that were cost-saving, both of which targeted overweight or obese children [38]. This demonstrates that there were several value-for-money treatment options for overweight and obese children and adults in Australia. The exceptions were the Weight Watchers program and the use of pharmacotherapy for obesity treatment in adults, which were not cost-effective with ICERs of approximately AUD 84,000 and AUD 200,000 per DALY averted respectively [39,48]. The pharmacotherapy results for adults differed to the results for orlistat in adolescents, which reported a net ICER of approximately AUD 8,000 per DALY [38]. This reflects the different target groups (adults verses adolescents) and the differing assumptions made by the respective ACE Working Groups on decay effects and the associated regain of weight loss [48].

**Table 1:** Cost-effectiveness of primary prevention *versus* treatment (and secondary prevention) interventions from the Assessing Cost-Effectiveness (ACE)-Obesity and ACE-Prevention studies.

Cost-effectiveness	Intervention Classification ^≠^	
Cost-saving **^†^**	**Treatment (and Secondary Prevention)**	**Primary Prevention**
	Family-based General Practitioner (GP) programme targeted at obese children [38] Multi-faceted targeted school-based programme for overweight and obese children [38]	School (curriculum)-based education programme to reduce television viewing [38] Multi-faceted school (curriculum)-based programme including nutrition and physical activity [38] School-based education programme to reduce sugar-sweetened drink consumption [38] Reduction of advertising of unhealthy food and beverages to children [43] Front-of-pack traffic light nutrition labelling [47] Unhealthy food and beverage tax (10%) [47]
Cost-effective **^‡^** (ICER ≤ $50,000/DALY)	Orlistat (obese adolescents **^µ^**) [38] Family-based GP programme targeted at overweight and moderately obese children [45] Laparoscopic adjustable gastric banding (LAGB) for obese adults (BMI > 35) **^β^** [42] LAGB for obese adolescents [40] Diet and exercise for adults BMI > 25 [41] Low-fat diet for adults BMI > 25 [41]	Multi-faceted school (curriculum)-based programme without an active physical activity component [38]
Not cost-effective **^∫^** (ICER > $50,000/DALY)	Sibutramine (obese adults) * [48] Orlistat (obese adults) [48] Weight watchers [39]	Walking school bus [44] TravelSMART schools **^α^** [46] Active after schools communities program [49] Lighten up to a healthy lifestyle weight-loss programme for adults **^π^** [39]

Notes: ^**≠**^ The costs included in the economic analyses are the costs of intervention implementation, delivery and the healthcare ramification costs or cost offsets. Productivity costs have not been included. **^†^** Interventions with net cost-effectiveness results (includes cost offsets) which are cost-saving. **^‡^** Interventions with incremental cost-effectiveness ratios (ICER) below the threshold value of AUD 50,000 per DALY averted. **^∫^** Interventions with ICER above the threshold value of AUD 50,000 per DALY averted. **^µ^** Orlistat is restricted for use in adults only in Australia. **^β^** Results were cost-saving when the intervention was targeted at obese adults with BMI > 40. * Withdrawn from Australian in 2010. **^α^** School and community based intervention aimed to increase active transport. **^π^** Although not restricted/targeted, the majority of participants were overweight or obese.

**Table 2:** Cost-effectiveness of program *versus* policy interventions from the Assessing Cost-Effectiveness (ACE)-Obesity and ACE-Prevention studies.

Cost-effectiveness	Intervention Classification ^≠^	
Cost-saving **^†^**	**Program**	**Policy**
	Family-based GP programme targeted at obese children [38] School-based education programme to reduce sugar-sweetened drink consumption [38] Multi-faceted targeted school-based programme for overweight and obese children [38]	School (curriculum)-based education programme to reduce television viewing [38] Multi-faceted school (curriculum)-based programme including nutrition and physical activity [38] Reduction of advertising of unhealthy food and beverages to children [43] Front-of-pack traffic light nutrition labelling [47] Unhealthy food and beverage tax (10%) [47]
Cost-effective **^‡^** (ICER ≤ $50,000/DALY)	Family-based GP programme targeted at overweight and moderately obese children [45] Diet and exercise for adults with Body Mass Index (BMI) > 25 [41] Low-fat diet for adults BMI > 25 [41]	Multi-faceted school (curriculum)-based programme without an active physical activity component [38]
Not cost-effective ^∫^ (ICER>$50,000/DALY)	Walking school bus [44] TravelSMART schools **^α^** [46] Active after schools communities program [49] Lighten up to a healthy lifestyle weight-loss programme for adults **^π^** [39] Weight watchers [39]	

Notes: ^**≠**^ The costs included in the economic analyses are the costs of intervention implementation, delivery and the healthcare ramification costs or cost offsets. Productivity costs have not been included. Individualised treatment interventions (e.g., Laparoscopic adjustable gastric banding and pharmacotherapy) have not been included in this table. **^†^** Interventions with net cost-effectiveness results (includes cost offsets) which are cost-saving. **^‡^** Interventions with incremental cost-effectiveness ratios (ICER) below the threshold value of AUD 50,000 per DALY averted. **^∫^** Interventions with ICER above the threshold value of AUD 50,000 per DALY averted. ^α^ School and community based intervention aimed to increase active transport. **^π^** Although not restricted/targeted, the majority of participants were overweight or obese.

On the other hand, the majority of primary preventive interventions in Table 1 targeting both children and adults were shown to be cost-saving. This suggests that, compared to treatment, preventive interventions were better options for the management of obesity in the long term. However there were also non cost-effective preventive interventions. Factors that resulted in preventive interventions being non cost-effective included the design of interventions not being specifically aimed at obesity prevention—for example, the active transport based interventions were designed to increase the number of children walking to school and to provide a safer traffic environment around schools [44,46]. This highlights a limitation of the application of the ACE methodology in ACE-Obesity and ACE-Prevention, where the benefits of an intervention independent of changes in weight were not captured [50].

**Table 3:** Cost-effectiveness of interventions aimed at food intake and physical activity from the Assessing Cost-Effectiveness (ACE)-Obesity and ACE-Prevention studies.

Cost-effectiveness		Intervention Classification ^≠^		
Cost-saving **^†^**	**Food Intake**		**Physical Activity**	**Food Intake & Physical Activity**
	School-based education programme to reduce sugar-sweetened drink consumption [38] Reduction of advertising of unhealthy food and beverages to children [43] Front-of-pack traffic light nutrition labelling [47] Unhealthy food and beverage tax (10%) [47]			School (curriculum)-based education programme to reduce television viewing [38] Multi-faceted school (curriculum)-based programme including nutrition and physical activity [38] Family-based GP programme targeted at obese children [38] Multi-faceted targeted school-based programme for overweight and obese children [38]
Cost-effective **^‡^** (ICER ≤ $50,000/DALY)	Orlistat (obese adolescents **^µ^**) [38] Laparoscopic adjustable gastric for obese adults (BMI > 35) **^β^** [42] Laparoscopic adjustable gastric for obese adolescents [40] Low-fat diet for adults BMI > 25 [41]			Family-based GP programme targeted at overweight and moderately obese children [45] Multi-faceted school (curriculum)-based programme without an active physical activity component (this intervention included education on physical activity) [38] Diet and exercise for adults BMI > 25 [41]
Not cost-effective **^∫^** (ICER > $50,000/DALY)	Sibutramine (obese adults) * [48] Orlistat (obese adults) [48]		Walking school bus [44] TravelSMART schools ^α^ [46] Active after schools communities program [49]	Lighten up to a healthy lifestyle weight-loss programme for adults **^π^** [39] Weight Watchers [39]

Notes: ^**≠**^ The costs included in the economic analyses are the costs of intervention implementation, delivery and the healthcare ramification costs or cost offsets. Productivity costs have not been included. **^†^** Interventions with net cost-effectiveness results (includes cost offsets) which are cost-saving. **^‡^** Interventions with incremental cost-effectiveness ratios (ICER) below the threshold value of AUD 50,000 per DALY averted. **^∫^** Interventions with ICER above the threshold value of AUD 50,000 per DALY averted. **^µ^** Orlistat is restricted for use in adults only in Australia. **^β^** Results were cost-saving when the intervention was targeted at obese adults with BMI > 40. * Withdrawn from Australian in 2010. **^α^** School and community based intervention aimed to increase active transport. **^π^** Although not restricted/targeted, the majority of participants were overweight or obese.

### 4.2. Program vs. Policy

The ACE interventions can also be classified as program or policy. Although the distinction between these two is not clear by definition, for practical purposes, policies can be defined by the policy instruments available to governments such as laws, regulations, taxes and services [51]. In contrast, programs can be defined as locally co-ordinated efforts implemented in specific sectors which are additional to the usual business of those sectors. Using this definition, the implementation of programs usually entails additional skills, services and funding.

The majority of policy interventions were cost-saving and all of the policy interventions evaluated had a net ICER below AUD 6,000 per DALY, well below the accepted cost-effectiveness threshold [38]. On the other hand, the program based interventions ranged from being cost-saving to being not cost-effective (Table 2). Policy interventions were usually more sustainable than programs. Programs were generally reliant on ongoing support funding, and therefore their long term sustainability influenced their cost-effectiveness profile. The affordability of programs is also dependent on the budget constraints of the program setting. For example, school based interventions which utilised regular teachers were more affordable and sustainable, and once implemented in the curriculum could be considered a policy rather than a program that required ongoing support by specialised staff.

### 4.3. Food vs. Physical Activity

With the aim of preventing or treating obesity, interventions can either have an impact on calorie intake, expenditure or both. Table 3 shows the ACE interventions categorised according to their impact on the different sides of the “energy balance equation”. The majority of interventions that targeted food intake only (80%) or both food intake and physical activity (78%) were either cost-effective or cost-saving. On the other hand, interventions which only targeted physical activity were found to be not cost-effective. This is likely related to the limited number and scope of the specific interventions related to physical activity evaluated in the previous ACE projects. Most commonly, policy interventions targeting physical activity are more difficult to evaluate due to the difficulty in gathering conclusive evidence on effectiveness.

The majority of physical activity targeted interventions from ACE-Obesity were in the school setting and required additional staff for implementation which affected the sustainability and cost-effectiveness profile [44,46,49]. However, there is some preliminary evidence from a current American priority setting study, which indicates that certain school based policy interventions targeting the intensity of physical activity can be cost-saving when implemented without the need for additional resources [52].

Another limitation of the physical activity interventions evaluated in the ACE projects was the lack of built environment interventions designed to encourage more physical activity (e.g., bike paths). Despite their limitations, the results from ACE-Obesity and ACE-Prevention are consistent with the view that the food environment is a key driver and has a greater impact on the development and therefore the management of obesity compared to the physical activity environment which is a moderator of the drivers of the obesity epidemic [1].

The cost-effectiveness results from the ACE projects from Table 1, Table 2 and Table 3 indicate that there are several value-for-money options for the management of obesity; however to date, there is limited evidence of implementation of even the most cost-effective interventions. A review of the “second stage filters” from these studies demonstrates that the majority of these interventions had significant impacts on the equity, acceptability and/or feasibility filters [38,43]. This reinforces the need to create a strong “political will for change” if interventions that are currently deemed not acceptable to influential stakeholders are to be implemented. Another reason for lack of policy action on cost-effective interventions could be the “partial analysis” methodology of the ACE evaluations (*i.e*., only health related outcomes were considered) [51]. Results from partial analyses may be less compelling to decision makers, particularly those outside the health care sector.

### 4.4. Other Priority Setting Studies in Obesity Prevention

There have been several other examples of priority setting of obesity interventions. Within the broader OPIC project on adolescent obesity in the Pacific region, a priority setting exercise was undertaken with the aim of facilitating evidence-based decision making by policy makers in Fiji and Tonga [53]. The study specifically focused on food policies that were specific to the local context, and modelled the impact of these policies on deaths averted due to non-communicable diseases. In the Fijian context, the five most cost-effective options were fiscal policies related to import duties and value-added-tax. In Tonga, the most cost-effective options were also predominantly related to food prices but also included policies related to food availability. Some of the interventions that were prioritised in the OPIC study have been translated into national policy and are in the process of being implemented. The key lessons from this priority setting study are the importance of considering the local food environment and the decision making context when prioritising obesity management interventions [53].

The OECD and the WHO developed a micro-simulation model to assess the cost-effectiveness of seven preventive interventions addressing behavioural risk factors linked with obesity (school based health promotion interventions, worksite health promotion interventions, mass media health promotion campaigns, fiscal measures to increase the cost of undesirable foods and decrease the cost of desirable foods, physician counselling of patients at high risk, the regulation of food advertising to children, and compulsory food labelling) [54]. The interventions were assessed against a comparator situation where only treatment interventions and no preventive interventions were available in six developing nations (Brazil, China, India, Mexico, Russia and South Africa; England was included for comparison) [54]. The interventions which were cost-effective (or cost-saving) across all countries were fiscal measures and food labelling over the 20 year time horizon; with food advertising joining this group over the 50 year time horizon. The authors comment that the characteristics of the more cost-effective interventions were that they had a greater coverage of the population and were relatively low cost to implement compared to the more targeted interventions assessed [54]. Two further studies using the ACE methodology for priority setting are currently being conducted in the USA (CHOICES project) and New Zealand (NZ-ACE), however final results from these studies are not yet available [55,56].

## 5. Review of the Evidence Gaps

**Figure 1:**
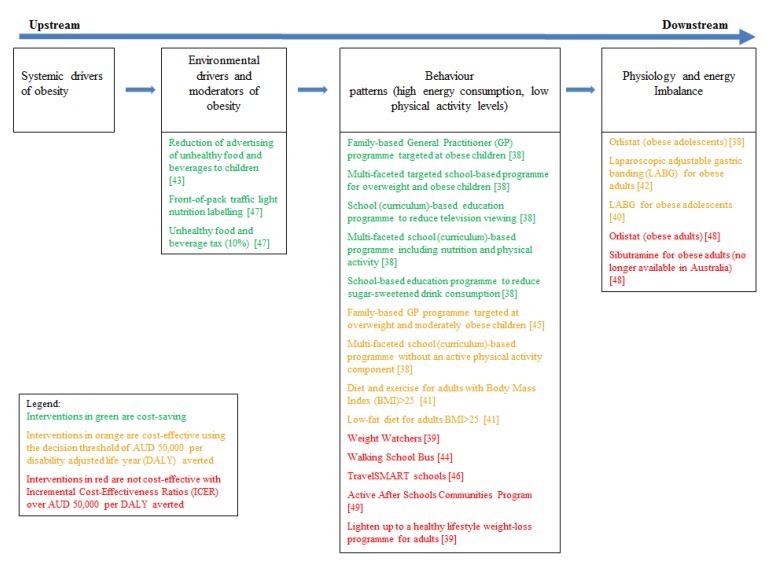
Interventions from the Assessing Cost Effectiveness (ACE) Obesity and ACE-Prevention studies, mapped to the continuum of obesity determinants and solutions.

To determine the current evidence gaps and to determine whether upstream or downstream interventions are the most cost-effective, the ACE interventions have been mapped to the continuum of the key determinants and solutions to the obesity epidemic (Figure 1). This continuum spans across upstream factors related to economic systems affecting nations to downstream factors affecting the physiology of individuals [1,10,57]. All of the treatment and the majority of the preventive interventions investigated thus far are downstream solutions that target predominantly individual behaviour and physiology. Amongst the preventive interventions, a further distinction can be made between health promotion interventions that target individual behaviour and those that target the obesogenic environment. The three preventive interventions that targeted the obesogenic environment (reduction of advertising of unhealthy food and beverages to children, front-of-pack traffic light nutrition labelling and a 10% tax on unhealthy food and beverages) were all cost-saving. The key determinants of their favourable cost-effectiveness profile were the small health impact for the whole population resulting in significant DALYs saved, together with a relatively low cost of implementation [57]. Off-setting their economic credential, all these interventions rated high on the scale of political difficulty surrounding their implementation [1]. The interventions which were found to be most cost-effective from the OECD/WHO priority setting exercise [54] can be categorised as “environmental drivers” in Figure 1 and are consistent with the findings from the ACE studies.

Although the discipline of economics has contributed to a better understanding of the causes of the obesity epidemic and has provided some insights into potential cost-effective options for change, there remain substantial gaps in the evidence base. The current evidence available from previous ACE projects and other priority setting exercises related to obesity management suggests that the most effective and cost-effective interventions are upstream and target the drivers of the obesogenic environment; however the majority of interventions that have been investigated to date are downstream. Several obesity commentators [1,10,57] have emphasised the importance of addressing not only the environmental drivers of the obesogenic environment, but importantly focusing on the systemic drivers of the problem. However there has been very limited investigation of the impact of upstream social and economic policies such as taxation, trade, and regulation of the market [1].

Learning from the successes of policies related to tobacco control, obesity prevention will require the implementation of a range of interventions across several sectors. Although the health sector bares a substantial burden related to obesity, policy actions that may have the greatest impact on curtailing the obesity epidemic span multiple sectors, such as agriculture, finance, urban planning, and transport [57]. Furthermore, there is evidence that the general economy also bares a substantial burden related to obesity due to lost productivity and absenteeism [58,59,60]. It is therefore in the interests of multiple sectors to contribute to the solution. Despite this, there is limited effectiveness and cost-effectiveness evidence related to interventions in these sectors and there are several challenges involved in economic evaluation of upstream obesity prevention policy interventions across various non-health sectors. These relate to limited evidence of effectiveness; difficulties in capturing the benefits of an intervention across multiple sectors (e.g., public transport policies have an impact on traffic, but may also have a health impact related to increased physical activity); assessing the effectiveness of several interventions implemented concurrently; incorporating equity impacts into the technical economic analyses and aiding the translation of results into effective policy action.

A recent review of the evidence base for obesity prevention by the Institute of Medicine (USA) concluded that there was a striking contrast between the importance of addressing the issue of obesity and the lack of a knowledge base to inform decision making on prevention efforts [61]. Nonetheless, the paucity of evidence need not and should not preclude the making of allocative decisions; and if the discipline of economics is to play a significant role in this exercise, it is important that quality economic evaluations are undertaken based on the best evidence available which may not always equate to the best evidence possible [62]. In order to effectively evaluate interventions which do not have RCT evidence, traditional evidence hierarchy frameworks need to be expanded to incorporate a variety of secondary evidence sources such as “indirect” and “parallel” evidence. While initial work in this area has commenced [61,62], to date there are limited examples of how secondary evidence should be incorporated into economic evaluations.

The ACE studies provide very useful information, but the study perspective focussed on the health sector and didn’t capture the full range of benefits for interventions that span or involve other sectors. Methodological advancements in economic evaluation are required to capture the systems effects of preventive interventions and to capture the full range of inter-sectorial benefits. It is anticipated that cost-benefit analysis where all costs and benefits are measured in monetary terms holds promise for such evaluations.

In Australia and in many other developed nations, obesity is most prevalent amongst those in the most disadvantaged SES groups [63,64]. Decision makers need information on the equity impacts of obesity prevention interventions to understand the potential of policy interventions to broaden or narrow the health gap between the most advantaged and disadvantaged members of society. The previous ACE projects considered the equity impacts of interventions qualitatively alongside the cost-effectiveness results; however more research is required to ascertain the most appropriate methods available to incorporate equity impacts into the technical analyses of an obesity priority setting exercise.

Further adding to the complexity of evidence-based decision making in obesity prevention is the political difficulty involved in implementing interventions that affect the interests of the very powerful food industry [57,65]. Priority setting exercises which use a broad societal perspective and explicitly consider the financial impact of proposed interventions on the private sector may enhance the ability of the results to have a more significant impact on policy decision making. In addition, a key component of future priority setting exercises should be the engagement and collaboration with decision makers outside the health care sector throughout the evaluation process.

## 6. Conclusions

Economic factors, such as the focus on growth and increasing consumerism is driving increased consumption of foods, beverages and energy saving devices, and has produced an obesogenic environment where passive over-consumption is pervasive [1]. The economic case for government intervention in obesity prevention is strong. The discipline of health economics can assist in resource allocation decision making by providing high quality evidence on the cost-effectiveness of a range of obesity management interventions. Previous priority setting exercises have made progress in advancing the knowledge base related to value-for-money options to curtail the obesity epidemic. A review of these studies revealed that interventions aimed at primary prevention were likely to be more cost-effective compared to treatment or secondary prevention interventions. In addition, policy interventions were found to be more sustainable and therefore more cost-effective than programs which require ongoing funding for implementation. Finally, interventions targeting food intake (or food intake combined with physical activity) were likely to be more cost-effective than interventions only targeting physical activity. The interventions most likely to be cost-effective were the upstream interventions that targeted the environmental drivers of the obesity epidemic such as regulation related to unhealthy food and beverage advertising, front of pack nutrition labelling and taxes on unhealthy food and beverages. However, there are very few interventions that have been evaluated in a priority setting context which address the systemic drivers of obesity.

In order to make a more significant contribution to obesity prevention, future priority setting exercises need to focus on upstream interventions, advance current methodologies, and enhance the translation of evidence into effective public health policy. In this regards, it is reassuring that there is active research which will progress these areas. Examples from Australia include a research program led by Deakin University that is investigating the cost-effectiveness of 40 non-health sector interventions and aims to incorporate equity impacts into the cost-effectiveness methods [66]; a research group from the University of Melbourne investigating the cost-effectiveness of several interventions related to the built environment [67]; and complementary to both of these projects, is work being undertaken by a consortium led by the George Institute for Global Health around methodological advancements related to the evaluation of preventive interventions including the evaluation of systems effects.
